# Prediction of site-specific drug deposition via dry powder inhaler using non-invasive real-time particle emission signal monitoring system

**DOI:** 10.3389/fphar.2026.1774142

**Published:** 2026-02-27

**Authors:** Sakiko Hatazoe, Daiki Hira, Tetsuri Kondo, Yuki Shigetsura, Natsuki Imayoshi, Yurie Katsube, Keiko Ikuta, Yuki Kunitsu, Keisuke Umemura, Satoshi Hamada, Satoshi Ueshima, Shunsaku Nakagawa, Masahiro Tsuda, Susumu Sato, Mikio Kakumoto, Tomohiro Terada

**Affiliations:** 1 Department of Clinical Pharmacology and Therapeutics, Kyoto University Hospital, Kyoto, Japan; 2 Graduate School of Pharmaceutical Sciences, Kyoto University, Kyoto, Japan; 3 Department of Respiratory Medicine, Shonan Fujisawa Tokushukai Hospital, Fujisawa, Kanagawa, Japan; 4 Department of Respiratory Medicine, Graduate School of Medicine, Kyoto University, Kyoto, Japan; 5 Department of Advanced Medicine for Respiratory Failure, Graduate School of Medicine, Kyoto University, Kyoto, Japan; 6 College of Pharmaceutical Sciences, Ritsumeikan University, Shiga, Japan; 7 Department of Respiratory Care and Sleep Control Medicine, Graduate School of Medicine, Kyoto University, Kyoto, Japan

**Keywords:** dry powder inhaler, non-invasive, photo reflection method, real-time monitoring, site-specific drug deposition

## Abstract

**Background:**

Accurate evaluation of regional drug deposition within the respiratory tract is essential for optimizing inhalation therapy efficacy and minimizing adverse effects. However, non-invasive, real-time quantitative methods for site-specific drug delivery assessment remain limited.

**Objective:**

To develop mathematical models to predict site-specific drug deposition from a dry powder inhaler (DPI) using a non-invasive, real-time photo reflection method (PRM).

**Methods:**

Using Symbicort^®^ Turbuhaler^®^ as a model DPI, four inhalation patterns varying in peak flow rate (PFR: 30–60 L/min) and flow increase rate (FIR: 3.2–9.6 L/s^2^) were simulated using a human inhalation flow simulator. Aerodynamic particle deposition of budesonide was quantified as the fine particle fraction for the whole lung (FPF_WL_), peripheral airways (FPF_PA_), and oropharyngeal region using an Andersen Cascade Impactor. Particle emission signals were monitored via PRM. The relationship between particle emission signals and deposition performance was analyzed using four univariate models: linear, logarithmic, Hill, and Emax.

**Results:**

Increased PFR and FIR enhanced drug deposition in both the lungs and oropharyngeal region. FPF_WL_ and FPF_PA_ were strongly correlated with total particle emission intensity over time with the Hill model (*R* ^2^ = 0.86 and 0.74 for FPF_WL_ and FPF_PA_, respectively), reflecting nonlinear deagglomeration. Oropharyngeal deposition correlated with flow rate at particle emission peak, fitting a linear model (*R* ^2^ = 0.82), consistent with inertial impaction mechanisms.

**Conclusion:**

Using an *in-vitro* model, particle emission signals enable the prediction of site-specific drug deposition from DPI, providing non-invasive, real-time indices and offering personalized inhalation performance assessment beyond conventional flow rate metrics.

## 1 Introduction

To maximize the therapeutic efficacy of inhaled medications and minimize adverse effects, it is essential to assess not only the total administered dose but also the regional distribution of drug deposition within the lungs (Beinart et al., 2024; Lazarinis et al., 2024; Cottini et al., 2025). In patients with asthma and chronic obstructive pulmonary disease (COPD), adequate drug distribution to the peripheral airways has been reported to be significantly associated with improved symptom control, reduced exacerbation frequency, and long-term maintenance of lung function (Mastalerz and Kasperkiewicz, 2011; Usmani et al., 2021; Clarà et al., 2023; Darquenne et al., 2024). Conversely, excessive oropharyngeal deposition of inhaled corticosteroids (ICS) is known to cause local adverse events, such as oral candidiasis and dysphonia, and systemic side effects through gastrointestinal absorption following swallowing (Dubus et al., 2001; Kaur and Singh, 2018; Arevalo and Buban, 2023; Gümrü and Akkitap, 2022). These adverse effects can lead to reduced patient adherence, which is a critical issue in clinical practice (Engelkes et al., 2015; Asamoah-Boaheng et al., 2021). Therefore, a method for quantifying and optimizing site-specific deposition on an individual basis is highly desirable.

Inhalation therapy is the initial treatment for asthma and COPD (Global Initiative for Asthma - GINA, 2025; Global Initiative for Chronic Obstructive Lung Disease, 2025). By enabling the direct delivery of medications to target sites, this approach facilitates a rapid onset of action while reducing systemic exposure and associated adverse effects (Chandel et al., 2019; Wang et al., 2024). Dry powder inhalers (DPIs) are widely used in clinical settings owing to their superior physicochemical stability and ability to achieve efficient pulmonary deposition through patient-generated inspiratory flow (Dhoble et al., 2024; Islam et al., 2024). However, the actual dose and distribution of drugs delivered to the lungs can vary substantially owing to multiple factors, including respiratory function, inhalation flow profiles, device design, formulation properties, and operational errors (Borghardt et al., 2018; Haidl et al., 2016; Nováková et al., 2022).

Previous attempts to predict therapeutic efficacy have used plasma drug concentration monitoring (Alahmadi et al., 2021). Although correlations with clinical outcomes have been observed, such approaches are limited by considerable interpatient and inter-formulation variability, making it difficult to selectively quantify pulmonary drug deposition (Getz and Carroll, 2017). *In vivo* imaging techniques using radiolabeled drugs have enabled the quantification of regional deposition; however, their invasive nature and procedural complexity restrict their utility to regulatory studies rather than routine clinical practice (Meseguer et al., 1994; Newman et al., 2000). Therefore, non-invasive methods such as *in vitro* and *in silico* testing are being explored. These studies have substantially advanced our understanding of how inhalation maneuver and device–powder interactions affect aerosol generation and regional deposition. However, these approaches typically depend on predefined inhalation patterns and airway geometries (Rebello et al., 2022). There is limited visibility into day-to-day, patient specific inhalation behavior in real-world clinical practice, where technique errors and self-adapted maneuvers may occur. Although the development of digital health integrated smart inhalers is underway, there is as yet no established method to directly evaluate drug deposition by accounting for individual inhalation patterns and airway anatomy (Bosnic-Anticevich et al., 2023). Therefore, practical methods are required to assess site-specific drug delivery based on real-time and non-invasive indices obtained during actual patient inhalation.

The results of our previous study demonstrated that a particle emission signal derived from the photo reflection method (PRM) can serve as non-invasive, and real-time indices for total pulmonary drug deposition via Symbicort^®^ Turbuhaler^®^ (Hatazoe et al., 2024). In PRM, the reflected light is measured as the aerosol released from an inhaler passes through the laser sensing region. Symbicort^®^ Turbuhaler^®^ is widely used as a combination therapy of an inhaled corticosteroid (budesonide) and a long-acting beta2-agonist (formoterol fumarate) for the treatment of asthma and COPD. Symbicort^®^ is characterized as a formulation where micronized particles (1–5 μm) of the two active ingredients and lactose form soft agglomerates (Buttini et al., 2016). It has been reported that these budesonide and formoterol exhibit comparable aerodynamic behavior in terms of the fine particle fraction (FPF). However, because the therapeutic and adverse effects of inhaled drugs originate from drug deposition in different anatomical regions, a detailed analysis of drug deposition sites in the respiratory system is necessary. Therefore, the aim of the present study was to develop a predictive model capable of simultaneously estimating drug delivery to the peripheral airways, central airways, and oropharyngeal regions using an established PRM-based monitoring system.

## 2 Materials and methods

### 2.1 Materials

Symbicort^®^ Turbuhaler^®^ 60 doses (AstraZeneca K.K., Osaka, Japan; containing 160 μg of budesonide (BUD) and 4.5 μg of formoterol fumarate dihydrate (FOR) per dose) was used as a model dry powder inhaler. BUD was purchased from Tokyo Chemical Industry Co., Ltd. (Tokyo, Japan) as a standard sample for high-performance liquid chromatography (HPLC). Other reagents and solvents used were of analytical and HPLC grades, respectively.

### 2.2 *In vitro* testing of regional lung deposition using PRM

**FIGURE 1:**
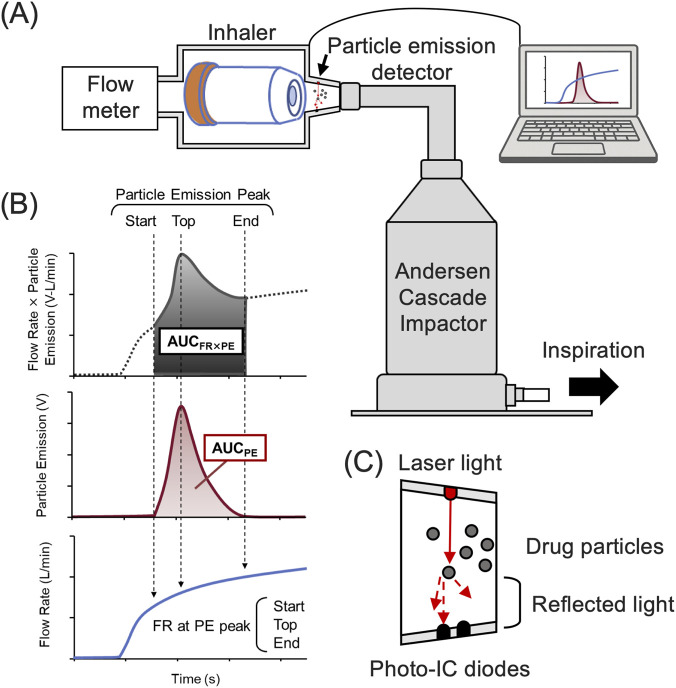
Schematic diagrams of the *in vitro* evaluation system using particle emission monitoring system. **(A)** The particle emission signal monitoring system was composed of particle emission signal detector with photo reflection method, a flow meter, and an Andersen Cascade Impactor (ACI). **(B)** Particle emission signal indices. **(C)** Particle emission signal detector. A laser irradiates particles passing through the section, and intensity of reflected light is measured with photo-IC diodes.

An *in vitro* system was established to assess the relationship between inhalation patterns and regional drug deposition using a combination of particle emission signal monitoring and cascade impaction analysis (Figure 1). Four inhalation patterns—Quick60, Slow60, Mild60, and Quick30—were defined based on varying peak flow rate (Defined PFR, 30–60 L/min) and flow increase rate (FIR, 3.2–9.6 L/s^2^), and reproduced using a human inhalation flow simulator (Hira et al., 2018). FIR was defined as the initial slope of the flow-time curve during the early inhalation phase and was controlled by a valve connected to a vacuum pump, as described previously (Hatazoe et al., 2024). Quick60 and Quick30 were generated with the rapid release of valve. By contrast, Slow60 was controlled with slower opening valve. Mild60 without valve operation, which resulted in a more gradual increase in flow rate despite a similar target PFR to Slow60. The inhalation duration was fixed at 5 s for all the patterns to specifically evaluate the impact of the early inhalation phase, given that this parameter is reported to be less influential on DPI performance than PFR or FIR (Hira et al., 2010). Three independent analyses were conducted for each inhalation pattern.

The particle emission signals were detected using a previously developed PRM-based monitoring system (Hatazoe et al., 2024). Particle emission signal was recorded using PRM by measuring laser light reflection caused by the aerosol passing through a fixed measurement section, simultaneously with inhalation flow rate. The following indices were calculated: peak value of particle emission (Peak PE, V); flow rate at the start, peak, and end of the particle emission signal (FR at PE peak start, top, and end, L/min); area under the time-particle emission curve (AUC_PE_, V-s); and area under the time product of particle emission and flow rate curve (AUC_FR×PE_, V-s-L/min). AUC_FR×PE_ was set as an index of the total drug amount released (Kondo et al., 2023).

The aerodynamic particle deposition of BUD was determined using an Andersen Cascade Impactor (ACI) (Sibata Scientific Technology Ltd., Tokyo, Japan). To avoid aerosol bounce, ACI was refrigerated for 1 h prior to inhalation. After inhalation, the BUD deposited on each stage of the ACI was collected by applying 10 mL of 20% ethanol. The concentration of BUD in each sample was quantified using HPLC-UV (Hatazoe et al., 2024). For evaluation of mass balance, the output efficacy (OE, %) was calculated (Equation 1). As indicators of therapeutic efficacy, the fine particle fraction for the whole lung (FPF_WL_, %) and peripheral airway deposition (FPF_PA_, %) were defined as the deposition after stage 3 (cutoff diameter <4.7 μm at 28.3 L/min) and stage 5 (cutoff diameter <2.1 μm at 28.3 L/min) of ACI, respectively (Equations 2, 3). In addition, depositions in the oral cavity and pharynx regions, which serve as indicators of potential adverse effects, were calculated as described in Equation 4. The theoretical released dose was indicated as the nominal dose of BUD (160 μg). This analysis was conducted to evaluate the shift in site-specific drug deposition by inhalation pattern using ACI stage groupings, rather than to determine aerodynamic particle size distribution.

$$\text{OE }\left(\%\right)=\frac{\left(\text{Mass}\;\text{recovered}\;\text{from}\;\text{ACI}\right)}{\left(\text{Theoretical}\;\text{released}\;\text{dose}\right)}\times 100$$ (1)

$${\text{FPF}}_{\text{WL}}\;\left(\%\right)=\frac{\left(\text{Mass}\;\text{recovered}\;\text{on}\;\text{and}\;\text{after}\;\text{stage}\;3\right)}{\left(\text{Theoretical}\;\text{released}\;\text{dose}\right)}\times 100$$ (2)

$${\text{FPF}}_{\text{PA}}\;\left(\%\right)=\frac{\left(\text{Mass}\;\text{recovered}\;\text{on}\;\text{and}\;\text{after}\;\text{stage}\;5\right)}{\left(\text{Theoretical}\;\text{released}\;\text{dose}\right)}\times 100$$ (3)

$$\text{Oral}-\text{Pharynx }\left(\%\right)=\frac{\left(\text{Mass}\;\text{recovered}\;\text{from}\;\text{Induction}\;\text{port}\;\text{to}\;\text{stage}\;2\right)}{\left(\text{Theoretical}\;\text{released}\;\text{dose}\right)}\times 100$$ (4)

### 2.3 Morphological analysis of particles of Symbicort^®^ Turbuhaler^®^

Field-emission scanning electron microscopy (FE-SEM; JSM-7900F; JEOL Ltd., Tokyo, Japan) was used to visualize the morphology of dispersed drug particles. Samples included drugs contained within the Symbicort^®^ Turbuhaler^®^ device before inhalation and particles collected from ACI stages 0, 3, and 5 under Quick60 and Quick30 inhalation conditions. Samples on the specimen mounts were coated with osmium before imaging (Neoc-Pro/P, Meiwafosis Co., Ltd., Tokyo, Japan).

The geometric diameters of the particles that were deagglomerated and deposited on ACI Stage 3 after the inhalation of Quick 60 were determined using AI-based particle size analysis software (GeXeL, AI-number: STD-00048, KNiT Inc., Osaka, Japan). The volume-weighted size distribution was computed, and D_10_, D_50_, D_90_ values were calculated from the cumulative volume distribution.

### 2.4 Construction and evaluation of predictive models

Univariate regression analyses were conducted to investigate the relationships between site-specific drug deposition and inhalation parameters or particle emission indices. Four mathematical models were tested: linear, logarithmic, Hill, and Emax models, as shown in Equations 5–8. Parameters were estimated by fitting a curve using linear or nonlinear least squares regression with R version 4.5.0 (R Foundation for Statistical Computing, Vienna, Austria).

y=ax+b (5)

$$y=\log\left(x\right)+b$$ (6)

$$y=\frac{D_{\max}\times x^{N}}{{\text{DS}}_{50}^{N}+x^{N}}$$ (7)

$$y=\frac{D_{\max}\times x}{{\text{DS}}_{50}+x}$$ (8)

In these models, y denotes the deposition fraction at a specific respiratory tract site, and x represents an explanatory variable derived from inhalation flow rate or particle emission indices. The parameter D_max_ refers to the maximum deposition achievable. DS_50_ is defined as the critical level of shear stress or flow energy required to achieve 50% of D_max_, effectively serving as an index of the threshold beyond which particle disintegration and dispersion are substantially accelerated. The exponent N characterizes the steepness of the transition in deposition, reflecting the degree of nonlinearity and structural abruptness in the deagglomeration process, such as the layer-by-layer collapse of particle agglomerates.

The model with the highest coefficient of determination (*R* ^2^) was selected as the optimal model for each target variable. Model validity and predictive accuracy were assessed using the Akaike Information Criterion (AIC), root mean square error (RMSE), and mean absolute error (MAE). These parameters are defined in Equations 9–11:

$$\text{AIC}=2k-2\ln\left(L\right)$$ (9)

$$\text{MAE}=\Sigma \left|y_{i}-\hat{y_{i}}\right|/n$$ (10)

RMSE=Σyi−yi^2n (11)

where *k* is the number of model parameters, and *L* is the likelihood function (Akaike, 1974). Additionally, yᵢ and ŷᵢ denote the observed and predicted values, respectively, and *n* is the number of observations (Chai et al., 2014).

Comparisons among inhalation patterns were performed using one-way analysis of variance (ANOVA), followed by Tukey’s multiple comparison test. The level of significance was set at *p* < 0.05. All analyses were conducted using R version 4.5.0 (R Foundation for Statistical Computing, Vienna, Austria).

## 3 Results

The profiles of the inhalation flow parameters and particle emission signal indices for each inhalation pattern are summarized in Table 1. As PFR and FIR increased, both Peak PE and AUC_FR×PE_ showed a corresponding increase, with the highest values observed under the Quick60 condition, which featured the most rapid increase in flow rate.

**TABLE 1:** Inhalation flow parameters and particle emission signal indices under four different inhalation patterns (n = 3, mean ± SD).

Inhalation pattern	Quick60	Slow60	Mild60	Quick30
Actual PFR (L/min)	56.7 ± 0.44	55.7 ± 0.40	56.0 ± 0.04	27.7 ± 0.31
Defined PFR (L/min)	60	60	60	30
FIR (L/s^2^)	9.3 ± 0.7	4.7 ± 0.1	4.0 ± 0.4	3.4 ± 0.1
Peak PE (V)	2.89 ± 0.39	1.44 ± 0.24	1.44 ± 0.07	1.10 ± 0.18
AUC_FR×PE_ (V.s.L/min)	6.78 ± 0.66	4.95 ± 0.73	3.83 ± 0.65	2.08 ± 0.30
FR at PE peak top (L/min)	37.7 ± 0.6	33.6 ± 0.5	23.2 ± 1.7	18.3 ± 0.5
FR at PE peak end (L/min)	46.5 ± 0.8	45.3 ± 0.2	36.6 ± 1.7	20.8 ± 0.3

Data are presented as mean ± standard deviation (n = 3). AUC, area under the curve; PFR, peak flow rate; FR, flow rate; PE, particle emission.

The inhalation performance parameters for all inhalation patterns are shown in Table 2. The OE of the Quick30 pattern was significantly lower than that of the other PFR60 patterns. Both the FPF_WL_ and FPF_PA_ increased with higher PFR and FIR, ranging from 15.2% to 57.2% and from 3.1% to 11.9%, respectively. Drug deposition in the oropharynx ranged from 10.5% to 36.3%, with higher values observed under Quick60 (*p* < 0.05 vs. Mild60 and Quick30).

**TABLE 2:** Inhalation performance for all inhalation patterns (n = 3, mean ± S.D.).

Inhalation pattern	Quick60	Slow60	Mild60	Quick30
OE (%)	83.9 ± 7.2	68.3 ± 9.6	62.5 ± 3.5	32.0 ± 5.0
Oral-pharynx (%)	31.9 ± 3.5	27.2 ± 4.2	19.5 ± 1.0	14.0 ± 2.6
FPFWL (%)	52.0 ± 3.8	41.0 ± 6.7	43.0 ± 2.4	18.0 ± 3.7
FPFPA (%)	10.9 ± 0.8	8.5 ± 0.8	10.1 ± 0.3	4.4 ± 1.0

OE, output efficacy represents mass balance, FPF_WL_, fine particle fraction deposited at or beyond ACI, Stage 3 (whole lung); FPF_PA_, fine particle fraction deposited at or beyond Stage 5 (peripheral airway). All indices are represented as a % nominal dose of budesonide (160 μg).

**FIGURE 2:**
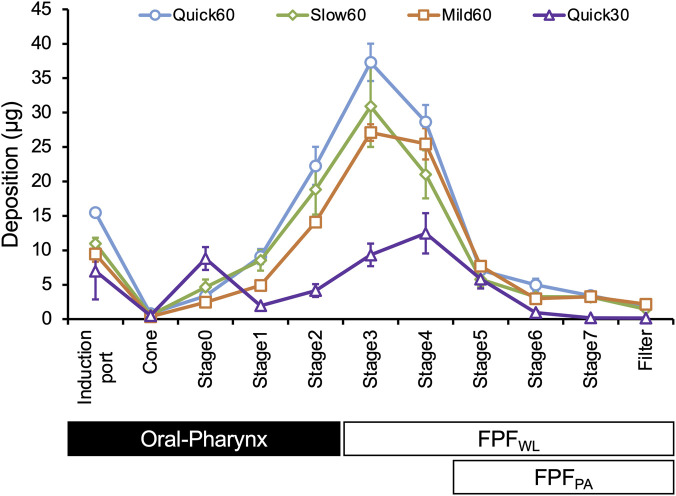
Drug deposition at each stage of ACI in four inhalation patterns (n = 3, mean ± S.D.) The amount of BUD deposition at each stage of ACI across all inhalation patterns is shown. The ranges of ACI stages corresponding to Oral-Pharynx, whole lung (FPF_WL_), and peripheral airway (FPF_PA_) are also indicated.

**FIGURE 3:**
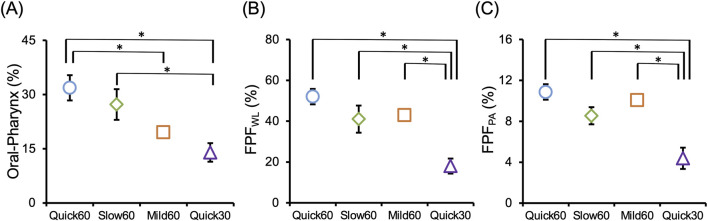
Influence of inhalation pattern on inhalation performance (n = 3, mean ± S.D.). Each panel shows the drug deposition in **(A)** Oral-Pharynx, **(B)** whole lung (FPF_WL_), and **(C)** peripheral airway (FPF_PA_). All indices are represented as a % nominal dose of budesonide (160 μg). *p < 0.05 (Tukey’s multiple comparison test).

The aerodynamic particle distributions obtained from ACI are shown in Figure 2. In the Quick30 condition, deposition at Stage 0—which corresponds to the pharyngeal region—was higher than that in the other patterns. Conversely, deposition in the induction port representing the oral cavity increased markedly with higher PFR and FIR. Three patterns with a defined PFR of 60 L/min exhibited peak deposition in Stage 3, corresponding to the central airways. By contrast, Quick30 showed a shift in peak deposition to a deeper region at Stage 4. These trends are reflected in Figure 3, which compares deposition indices across inhalation patterns, indicating that both pulmonary delivery and oropharyngeal deposition increased concurrently under high PFR and FIR conditions.

**FIGURE 4:**
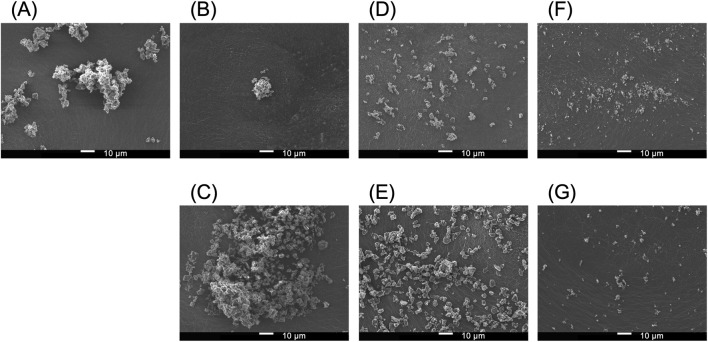
Scanning electron micrographs of particles collected from Symbicort^®^ Turbuhaler^®^ device and different ACI stages. **(A)** Undispersed aggregates inside the device before inhalation. Deposited particles at Stage 0 (pharynx region) under Quick60 **(B)** and Quick30 **(C)**, Stage 3 (central airway) under Quick60 **(D)** and Quick30 **(E)**, and Stage 5 (peripheral airway) under Quick60 **(F)** and Quick30 **(G)**. Scale bar, 10 µm.

**FIGURE 5:**
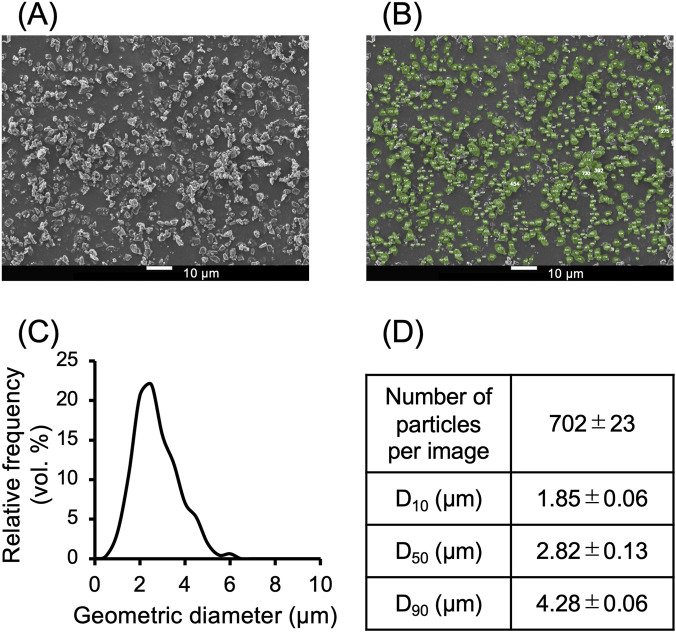
Geometric particle size distribution of particles deposited on ACI Stage 3 after inhalation of Quick60. **(A)** SEM images of particles deposited on ACI Stage 3 (Scale bar = 10 µm). **(B)** Particle images detected by the AI-based particle size analysis software GeXeL, with detected particles highlighted in green. **(C)** Histogram and **(D)** calculated parameters of the particle size distribution. Data are presented as mean ± standard deviation (n = 4).

SEM images of Symbicort^®^ Turbuhaler^®^ particles obtained before and after inhalation are shown in Figure 4. The undispersed aggregates were observed inside the device before inhalation (Figure 4A). Panels B–G show particles deposited on the stages 0 (pharynx region), Stage 3 (central airway) and Stage 5 (peripheral airway) of ACI, respectively, after a single inhalation. The upper images correspond to Quick60, and the lower images correspond to Quick30. Both inhalation patterns, aggregated drug particles larger than 10 μm were observed at stage 0. Partial disaggregation was evident in Stage 3, whereas Stage 5 showed well-dispersed fine particles. However, in Quick30, coarser agglomerates were observed in Stage 0. In addition, Figure 5 shows the geometric particle size distribution of particles deposited on Stage 3 using the AI-based particle size analysis. The image analysis appropriately identified the deagglomerated primary particles (Figures 5A,B). Furthermore, a sharp particle size distribution was confirmed, with a median particle diameter (D_50_) of 2.82 μm.

**FIGURE 6:**
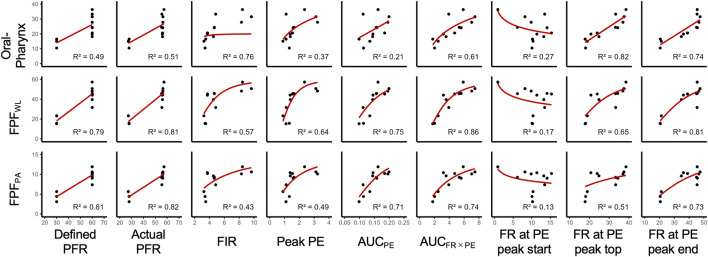
Regression models describing the relationship between inhalation performance and inhalation/particle emission parameters. Representative fits of the optimal models selected for predicting drug deposition in the oropharynx and lungs. Each black point represents the observed value in all inhalation patterns, and the red line indicates the regression model.

The results of the univariate regression analyses for model fitting are shown in Figure 6. In Table 3, we list the best-fit model types and associated equations for each inhalation performance. Furthermore, model evaluation metrics, including the AIC, RMSE, and MAE for all univariate regression models, are summarized in Table 4. In the case of FPF_WL_ and FPF_PA_, linear models with PFR exhibited strong correlations (*R* ^2^ = 0.81–0.82), whereas AUC_FR×PE_ showed a better fit with the Hill model, achieving the highest *R* ^2^ of 0.86 for FPF_WL_. In addition, a high correlation was confirmed between FR at PE peak end and the Hill model. By contrast, oropharyngeal indices were significantly predicted by the linear model with FR at PE peak top/end, and the Hill model with FIR. Whereas the logX model using AUC_FR×PE_ and linear model using PFR also showed moderate predictive power.

**TABLE 3:** Optimal univariate regression models for predicting inhalation performance parameters.

Object variable	Explanatory variables	Equation	Model	*R* ^2^	RMSE
Oral-pharynx	FR at PE peak top	$y\left(x\right)=-1.3+0.87x$	Linear	0.82	3.2%
	FIR	$y\left(x\right)=\frac{20\times x^{3}}{1.47^{3}+x^{3}}$	Hill	0.76	8.1%
	FR at PE peak end	$y\left(x\right)=-0.2+0.63x$	Linear	0.74	3.9%
FPF_WL_	AUC_FR×PE_	$y\left(x\right)=\frac{60\times x^{2.14}}{2.87^{2.14}+x^{2.14}}$	Hill	0.86	5.0%
	Actual PFR	$y\left(x\right)=-9.1+0.97x$	Linear	0.81	5.8%
	FR at PE peak end	$y\left(x\right)=\frac{60\times x^{2.72}}{27.9^{2.72}+x^{2.72}}$	Hill	0.81	5.8%
FPF_PA_	Actual PFR	$y\left(x\right)=-1.0+0.19x$	Linear	0.82	1.1%
	AUC_FR×PE_	$y\left(x\right)=\frac{15\times x^{1.39}}{3.24^{1.39}+x^{1.39}}$	Hill	0.74	1.4%
	FR at PE peak end	$y\left(x\right)=\frac{15\times x^{1.92}}{30.8^{1.92}+x^{1.92}}$	Hill	0.73	1.4%

For each outcome, the explanatory variable and corresponding prediction equation showing the highest model fit are listed. RMSE: root mean square error.

**TABLE 4:** Model fitting statistics, including *R* ^2^, AIC, RMSE, and MAE, for each explanatory and outcome pair.

*R* ^2^	Defined PFR	Actual PFR	FIR	Peak PE	AUC_PE_	AUC_FR×PE_	FR at PE peak start	FR at PE peak top	FR at PE peak end
Oral-pharynx	0.49	0.51	0.76	0.37	0.21	0.61	0.27	0.82	0.74
FPF_WL_	0.79	0.81	0.57	0.64	0.75	0.86	0.17	0.65	0.81
FPF_PA_	0.81	0.82	0.43	0.49	0.71	0.74	0.13	0.51	0.73
AIC									
Oral-pharynx	80.4	80.0	92.3	83.1	85.7	77.3	84.8	68.1	72.6
FPF_WL_	83.3	82.2	94.2	92.2	89.5	80.6	99.9	91.6	84.3
FPF_PA_	43.4	42.3	58.4	57.2	50.2	49.3	61.5	56.7	49.3
RMSE									
Oral-pharynx	5.4	5.3	8.1	6.0	6.7	4.7	6.4	3.2	3.9
FPF_WL_	6.07	5.80	8.78	8.07	7.22	4.99	12.10	7.87	5.82
FPF_PA_	1.15	1.10	1.98	1.88	1.40	1.35	2.45	2.00	1.35
MAE									
Oral-pharynx	4.6	4.5	6.4	4.2	4.9	3.8	5.1	2.8	3.5
FPF_WL_	4.34	4.24	7.53	6.85	5.69	3.90	10.28	6.09	3.96
FPF_PA_	0.90	0.86	1.67	1.56	1.25	1.20	2.04	1.69	1.14

Red cells indicate *R* ^2^ ≥ 0.80, and pink cells indicate *R* ^2^ ≥ 0.50. AIC: akaike information criterion; MAE: mean absolute error. Abbreviations as in Tables 1 and 2.

## 4 Discussion

We evaluated the impact of inhalation pattern on site-specific drug deposition and identified optimal predictors for each site in the respiratory tract. High PFR and FIR enhanced pulmonary drug delivery while also increasing oropharyngeal deposition. Univariate regression revealed that the optimal predictors differed by deposition site, indicating the need for site-specific evaluation in modeling inhalation performance.

DPIs are designed to contain agglomerated drug particles or drug-carrier complexes to avoid adhesion to internal surfaces. Turbulent energy from inhalation facilitates deagglomeration and generates fine particles with aerodynamic diameters suitable for peripheral airway delivery (Islam et al., 2024; Zheng et al., 2021). After drug release, these particles deposit through inertial impaction or gravitational sedimentation, exerting pharmacological effects (Demoly et al., 2014). This mechanism is particularly relevant for Turbuhaler^®^, which reportedly requires a PFR of at least 60 L/min to achieve adequate dispersion (Haidl et al., 2016). Consistent with this device feature, the overall emitted dose from Turbuhaler^®^ has been reported to increase with inhalation flow rate, typically reaching approximately from 40% to 80% (Farkas et al., 2023). In the present study, our OE showed a comparable flow rate dependency trend (Table 2). Moreover, FPF_WL_ and FPF_PA_ were significantly higher at 60 L/min than at 30 L/min, with the maximum FPF_WL_ observed being 57.2% under Quick60. SEM images (Figure 4) and particle size distribution analysis (Figure 5) showed dispersed particles at ACI Stages 3 and 5 under Quick60, whereas large agglomerates remained at Stage 0 under Quick30. Additionally, drug deposition at ACI Stage 0 was markedly higher under Quick30 than under the other inhalation patterns (Figure 2). These findings from SEM and ACI data collectively indicate that lower flow rates result in insufficient deagglomeration and the emission of coarse agglomerates, whereas high PFR facilitates progressive deagglomeration along the impactor stages and promotes distal lung delivery.

We found that the value of FPF_WL_ ranged from 15.2% to 57.2%, depending on the inhalation pattern. In previous *in vivo* studies with Turbuhaler^®^, investigators have reported pulmonary deposition values of 26.1% ± 10.5% in adult patients with asthma (Thorsson et al., 1998) and a range of 15.6%–47.2% in pediatric patients (Wildhaber et al., 1998). The lower end of the range of values in our study is consistent with these previous reports; however, the upper end exceeded what was previously reported. In clinical settings, 19%–78% of patients with asthma or COPD fail to achieve a target PFR of 60 L/min during DPI use (Broeders et al., 2001; Haikarainen et al., 2020; Bagherisadeghi et al., 2017; Weers, 2022). Therefore, ideal conditions, as in the Quick60 inhalation pattern, may not be replicable in real-world practice, contributing to differences between *in vitro* and *in vivo* findings.

The FPF_PA_ in this study ranged from 3.1% to 11.9%, which is lower than the reported 8.6%–24.8% range from *in silico* analyses of ICS delivery with Turbuhaler^®^ (Watz et al., 2021). This underestimation likely results from limitations of the ACI device. The ACI is designed for standard flow rates (28.3 L/min), and its aerodynamic cut-off diameters shift depending on the applied flow. At higher flow rates, enhanced inertial forces reduce the cut-off diameters of each stage, leading to premature capture of fine particles in upstream stages (Yoshida et al., 2017; Kamiya et al., 2009; Almeziny et al., 2025; van Holsbeke et al., 2018). In the present study, Oral-Pharynx increased under the higher PFR and FIR conditions (Figure 3A), and peak deposition shifted from Stage 3 to Stage 4 under Quick30 (Figure 2). These results suggest the occurrence of this inertial force impaction phenomenon. Conversely, because the deagglomeration of drug aggregates increases under high flow rate conditions, the Turbuhaler^®^ yielded higher FPF_PA_ results at higher flow rates (Figure 3C). As site-specific deposition of DPI formulations is determined by the balance between deagglomeration efficacy and inertial impaction, it is crucial to predict site-specific deposition across a wide range of inhalation patterns. Although this may lead to underestimation of peripheral delivery in absolute terms, the evaluation by ACI grouping remains valuable for assessing the relative influence of inhalation patterns on site-specific drug deposition.

Additionally, increased FIR was associated with elevated drug deposition in the oropharyngeal region, particularly under Quick60 conditions. Oropharyngeal deposition was higher at steeper flow acceleration, indicating that excessive FIR may enhance drug impaction in the upper airway. This phenomenon is consistent with the results of previous *in silico* studies that showed that particles in the 5–10 μm range are prone to inertial impaction at elevated flow rates (Sosnowski et al., 2006; Kadota et al., 2022). The oropharyngeal deposition we observed in the present study (10.5%–36.3%) was lower than that reported in previous *in vivo* studies with Turbuhaler^®^, where deposition to the oropharyngeal region ranged from 12.8% to 61.8% (Wildhaber et al., 1998; Hirst et al., 2001; Devadason et al., 1997). Such variability among studies is likely influenced by inter-individual differences in inhalation techniques, device handling, tongue positioning, and pharyngeal anatomy (Sosnowski et al., 2006; Xi and Yang, 2019). In the present study, we used the USP induction port (USP-IP), ACI cone and Stage 0 to Stage 2 as the surrogates for oropharyngeal regions. Although the USP-IP lacks anatomical fidelity, combining it with Stage 0 to Stage2 and applying cooling to reduce re-scattering (Zhou et al., 2011) enabled detection of flow-dependent differences in oropharyngeal deposition.

To identify the predictors of drug deposition, univariate regression analysis was performed using the inhalation flow rate and particle emission signal. Generally, for Turbuhaler^®^, FPF<5 µm increases with inspiratory flow rate, although several studies have reported that the incremental gain in FPF<5 µm diminishes at excessively high flow rates (Farkas et al., 2023). This behavior is considered to reflect a shift from disaggregation by shear stress within the device to enhanced inertial impaction in the upper airways. On this basis, we assumed that particle emission signal indices representing drug release may also exhibit nonlinear behavior. Accordingly, we adopted four different mathematical models (linear, logarithmic, Hill, and Emax) to capture such potential nonlinearities. As summarized in Table 3, pulmonary deposition indices (FPF_WL_ and FPF_PA_) correlated strongly with actual PFR, AUC_FR×PE_, and FR at PE peak end. These predictors reflect the intensity and duration of inhalation and drug release, suggesting that sustained inhalation efforts may be related to lung deposition. By contrast, oropharyngeal deposition showed strong correlation with the parameters related to the early phase of inhalation, namely, FR at PE peak top/end and FIR. Unlike time-averaged indices such as AUC_FR×PE_, FIR and FR at PE peak top/end are reflective of initial flow acceleration and the onset of turbulent airflow, both of which promote inertial impaction within proximal-airway structures.

Notably, AUC_FR×PE_ was a significant predictor for lung deposition. Symbicort^®^ Turbuhaler^®^ contains micronized drug particles blended with fine lactose particles of size <10 μm, which are loaded as large and soft agglomerates (∼0.5 mm in size) (Leung et al., 2015). Accordingly, the particle emission signals are likely to reflect light from both drug and lactose particles. As a result, even if lactose contributes to the particle emission signals, drug particle release involves a stepwise deagglomeration process that is dependent on the level of shear stress, making it difficult to describe the relationship using a simple linear model. Whether similar relationships between site-specific drug deposition and particle emission signals can be observed for DPIs with different formulation designs and deagglomeration mechanisms remains to be investigated.

By contrast, oropharyngeal deposition was best described by a linear model with FR at PE peak top. This likely reflects a process dominated by inertial impaction of particles on the upper airway walls, which increases proportionally with the magnitude of initial inhalation flow. In contrast to pulmonary deposition, which involves nonlinear processes such as stepwise deagglomeration, oropharyngeal deposition followed a more direct relationship in which the strength of inhalation was linearly associated with the amount of deposition. These findings highlight the need for site-specific model selection that captures the mechanisms underlying aerosol deposition, even when using the same predictors.

Traditional lung deposition predictions rely primarily on aerodynamic particle size and inhalation flow rates (Horváth et al., 2020; Longest and Holbrook, 2012; Kim and Hu, 2006). Although these emphasize airflow characteristics, they do not consider real-time drug release kinetics. For instance, profiles with comparable PFR can yield different aerosol release depending on the early phase inspiratory dynamics. In contrast, particle emission indices, such as AUC_FR×PE_, capture the aerosolization process in real time, improving the ability to potentially predict variability in site-specific deposition across individual inhalation patterns.

This study had several limitations. First, the present analysis was conducted using only Symbicort^®^ Turbuhaler^®^. Given the flow resistance, the amount and size of drug and lactose differences across inhalers, these models need to be validated with other inhalers. Second, we restricted our analysis to univariate regression models. The class of PFR and FIR was limited, and the intercorrelations among flow rate and PRM indices could lead to overfitting and unstable parameter estimates. Future studies with larger and more diverse inhalation patterns and devices will be required to develop multivariate prediction models. Finally, the clinical relevance of the particle emission signal remains unvalidated, and further human studies are warranted to establish external validity.

## 5 Conclusion

In this study, we demonstrated that regional drug deposition could be predicted based on inhalation pattern–dependent flow characteristics and particle emission signal measurements. Indicators reflecting inhalation intensity were effective in predicting pulmonary delivery, whereas parameters representing early-phase flow acceleration were more predictive of oropharyngeal deposition. These findings suggest that distinct factors govern the drug distribution across anatomical sites. Thus, compared to conventional assessments based on individual parameters such as flow rate, real-time flow and emission signals may serve as useful tools for assessing inhalation performance.

## Data Availability

The original contributions presented in the study are included in the article/supplementary material, further inquiries can be directed to the corresponding author.
